# Full Exploitation of Peach Palm (*Bactris gasipaes* Kunth): State of the Art and Perspectives

**DOI:** 10.3390/plants11223175

**Published:** 2022-11-21

**Authors:** Kamila de Cássia Spacki, Rúbia Carvalho Gomes Corrêa, Thaís Marques Uber, Lillian Barros, Isabel C. F. R. Ferreira, Rosely Aparecida Peralta, Regina de Fátima Peralta Muniz Moreira, Cristiane Vieira Helm, Edson Alves de Lima, Adelar Bracht, Rosane Marina Peralta

**Affiliations:** 1 Departamento de Bioquímica, Universidade Estadual de Maringá, Maringá 87020-900, Brazil; 2 Programa de Pós-Graduação em Tecnologias Limpas, Instituto Cesumar de Ciência, Tecnologia e Inovação—ICETI, Universidade Cesumar—UNICESUMAR, Maringá 87050-900, Brazil; 3 Centro de Investigação de Montanha (CIMO), Instituto Politécnico de Bragança, Campus de Santa Apolónia, 5300-253 Bragança, Portugal; 4 Laboratório Associado para a Sustentabilidade e Tecnologia em Regiões de Montanha (SusTEC), Instituto Politécnico de Bragança, Campus de Santa Apolónia, 5300-253 Bragança, Portugal; 5 Departamento de Química, Universidade Federal de Santa Catarina, Florianópolis 88040-900, Brazil; 6 Departamento de Engenharia Química, Universidade Federal de Santa Catarina, Florianópolis 88040-900, Brazil; 7 Embrapa Florestas, Colombo 83411-000, Brazil

**Keywords:** Amazon biome, sustainable agriculture, carotenoids, starch, circular economy, palm heart, sustainable food system, upcycling

## Abstract

The peach palm (*Bactris gasipaes* Kunth) is a palm tree native to the Amazon region, with plantations expanding to the Brazilian Southwest and South regions. This work is a critical review of historical, botanical, social, environmental, and nutritional aspects of edible and nonedible parts of the plant. In Brazil, the importance of the cultivation of *B. gasipaes* to produce palm heart has grown considerably, due to its advantages in relation to other palm species, such as precocity, rusticity and tillering. The last one is especially important, as it makes the exploitation of peach palm hearts, contrary to what happens with other palm tree species, a non-predatory practice. Of special interest are the recent efforts aiming at the valorization of the fruit as a source of carotenoids and starch. Further developments indicate that the *B. gasipaes* lignocellulosic wastes hold great potential for being upcycled into valuable biotechnological products such as prebiotics, enzymes, cellulose nanofibrils and high fiber flours. Clean technologies are protagonists of the recovery processes, ensuring the closure of the product’s life cycle in a “green” way. Future research should focus on expanding and making the recovery processes economically viable, which would be of great importance for stimulating the peach palm production chain.

## 1. Introduction

The peach palm (*Bactris gasipaes* Kunth) is a palm tree requiring a tropical climate that can be easily found throughout the Amazon region. Among the species of palm trees, the peach palm offers two food crops with commercial potential, the palm heart, which is recognized worldwide, and the fruits, typical of the Amazon region. The latter is widely consumed for breakfast, after cooking in water and salt, and in the manufacture of flours or culinary preparations [1,2,3,4]. The fruits can be orange, red or yellow and are commercially exploited as a source of starch and oil, the latter being rich in carotenoids [5,6,7].

Brazil generates approximately US$350 million annually from palm hearts, and it is estimated that the world market amounts to US$500 million, still with considerable growth potential. In addition to being considered the largest producer and consumer of palm hearts, the country is the world’s largest exporter of the commodity, accounting for approximately 95% of all palm hearts consumed in the world. Of these, 90% are of extractive origin from açaí (*Euterpes oleracea* Mart.), native to the Amazon Forest, and from juçara (*Euterpes edulis* Mart.), native to the Atlantic Forest [8]. Despite the economic index of the product being quite significant, the extraction of these species has contributed to endangering them, especially the juçara palm, which dies after the palm heart is harvested. Therefore, the rational use of other native palm trees to produce palm hearts, such as peach palms, has been promoted as a strategy to reduce the pressure of exploration on *Euterpe edulis* Mart. (juçara) and *Euterpe oleracea* Mart. (açaí) [8].

Although the cultivation of palm hearts is profitable for agribusinesses, the accumulation of waste generated after the extraction of the palm heart can reach 80–90% of its gross weight. The residues are usually exposed at the harvest site, and a large part is characterized as a fibrous material with a slow degradation rate, thus considered an environmental liability. As an alternative for valuing these residues, it has been suggested that they can be upcycled into novel products with high added value [9,10]. The definition of upcycling is the “reuse (of discarded materials) in such a way as to create a product of higher quality or value than the original” [11]. In other words, it means the repurposing of such materials, attributing them with a distinct function not foreseen in advance and which upgrades them [12].

Given this context, this review article aimed at addressing the history of the peach palm species *Bactris gasipaes* Kunth, its main characteristics, the use of both fruits and palm heart and the waste that is generated upon its exploration. Detailing these topics will provide useful basic information for investigators interested in developing new methods and new technologies related to the peach palm tree. Scientific articles and book chapters published mainly during the last 10 years (2012 to now) were selected. The databases Science Direct, PubMed, Scopus, Web of Science and Google Scholar were used for the advanced search performed with combinations of the key-words “*Bactris gasipaes* Kunth,” “pupunha,” “lignocellulosic residues,” “peach palm,” and “peach palm wastes.”

## 2. History of Peach Palm (*Bactris gasipaes* Kunth)

The Amazon biome is constituted by a humid and dense tropical forest with a hot and humid climate characterized by heavy rains that frequently occur throughout the year. Due to these factors, the Amazon is considered the habitat of several species that are commonly utilized and consumed by the local population and, therefore, easily found in local markets [13].

The palm trees have a pantropical distribution and presumed origins in the Tropical Forest. Their diversification began over the period of the Mesozoic era, about 100 million years ago [14,15]. The species *Bactris gasipaes* Kunth was one of the first palm trees to be domesticated for logging by the indigenous people. This happened in pre-Columbian times in the Southwestern region of the Amazon. The fruits of the species came to be used as an oil source [16]. Harvested fruits became part of the diet of the local population, usually from the North of Brazil, while palm hearts started to be consumed as a source of fiber [17,18].

## 3. Botanical and Morphological Aspects of *Bactris gasipaes* Kunth

The peach palm is a palm tree belonging to the Arecaceae/Palmae family, originating in the Amazon, and domesticated by the first peoples for its fruits, whose genetic differences are influenced by the place of cultivation, and which have different sensory characteristics, such as flavor and color. Although the family has many species, the most cultivated is *Bactris gasipaes* Kunth. This plant is characterized by being evergreen, with an erect stem and diameter ranging between 15 and 30 cm, reaching 15–20 m in height and, in some cases, up to 25 m [19,20,21].

**Figure 1:**
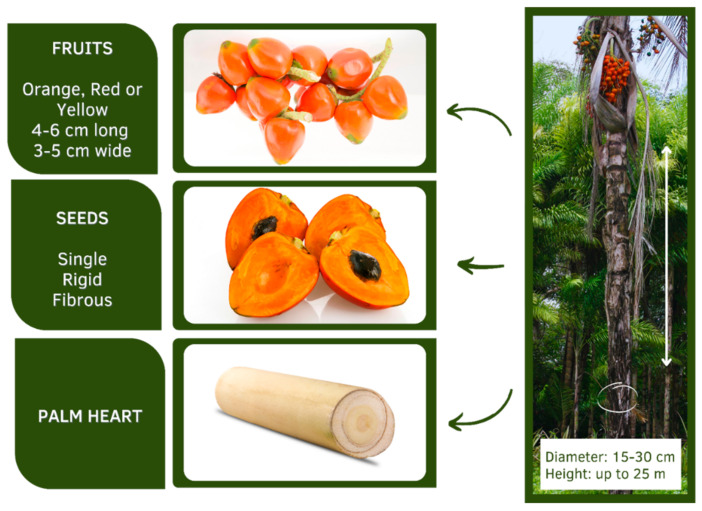
The peach palm tree and its commercially exploited parts (products), namely fruits, seeds, and palm hearts.

The peach palm fruit can be orange, red or yellow, is 4–6 cm long and 3–5 cm wide, and is composed of an edible pulp wrapped around a single rigid and fibrous seed (Figure 1). Fruits have different shapes, such as conical, ovoid, or ellipsoid, while the weight can vary from 20 to 205 g. The thin rind adhered to the pulp presents a diversified color (red, orange or yellow), a characteristic that depends on the variety and on the ripening stage of the fruit. The pulp is colorless, fleshy, and starchy/oily, with a mild bittersweet flavor and represents about 72% of the fruit’s weight, followed by the seeds (21%) and the skin (6%) [5,13,22].

The production of fruits is 5 to 10 bunches per year/plant, and each bunch can contain up to 12 kg of fruit [17]. Moreover, it is estimated that the harvest of 1 hectare can yield 10 tons of fruit per year. Considering that the peach palm market is consolidating in Brazil, it is necessary to have fruits to generate the seeds required by the large commercial plantations that are designed to meet the demand for palm hearts. This produces an excess of fruits that have already been used for the development of new products with added value [2].

**Figure 2:**
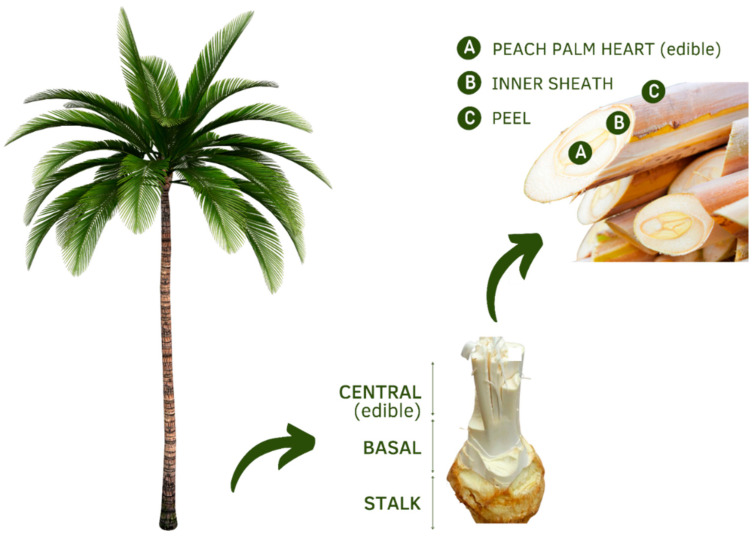
*Bactris gasipaes* palm stalk with its parts identified.

Figure 2 represents the *B. gasipaes* palm stalk with its parts identified. The stalk contains the palm heart, which is protected by two external wrappings and two internal ones. After harvesting, the two external wrappings are removed so that the palm heart remains protected by the two internal ones [23]. Approximately 59.6% of the total weight is represented by the hems, 14.6% by the basal part, 10.7% by the open leaves or “tip,” and only 15% of the palm heart is usable as food [8].

The peach palm tree has several advantages as a palm heart producer, which can be summarized as follows. The first one is precocity, as the tree starts to produce palm hearts 18 months after planting. Following comes the plant’s hardiness, vigor and productivity, which are characteristics appreciated by rural producers. Still more relevant is the fact that after extraction, the plant continues to emit new shoots and that this occurs continuously over a period of approximately 10 years. One must equally emphasize the quality of the palm heart since the species does not quickly darken after cutting. Finally, one must equally consider the possibility of a natural sale, with greater added value and ease in cultural treatments [8].

## 4. Edible Parts of the Peach Palm: Palm Heart and Fruits

The species *Bactris gasipaes* has, in principle, two edible parts, palm heart and fruit [1,4] (Figure 1). The peach palm tree can be cultivated for two different purposes: palm heart and fruit (popularly known as peach palm) production [1,24]. In the Brazilian Amazon, the peach palm tree is almost exclusively cultivated for fruit production purposes [13,25]. On the other hand, in the South and Southeast of Brazil, the peach palm tree is almost exclusively cultivated for palm heart production purposes, and the fruits are considered waste, being practically underexplored in the national and worldwide scenario [26].

### 4.1. Peach Palm Fruit

The peach palm fruit, in terms of its nutritional characteristics, is one of the most balanced among tropical fruits [27]. The peach palm fruit is of high quality due to its variable characteristics of chemical composition, genetic diversity, and yield, and it can be used in human and animal diets due to its high nutritional and energetic values [28].

The fruit can be consumed after cooking, being the main form of consumption by inhabitants of the Amazon, which is justified by the presence of significant amounts of calcium oxalate in the pulp and skin that causes a stinging sensation in the tongue, which is attenuated by cooking [29]. The ingestion of even small amounts of the *in natura* fruit is sufficient to cause an intense burning sensation in the mouth and throat, swelling of the airways and suffocation. If ingested in large amounts, it may even be fatal. Recovery from poisoning by large doses of calcium oxalate is possible; however, the liver and kidneys are permanently affected [1].

The peach palm fruit presents high nutritional value, rich in protein and fiber [30,31]. It has a high content of carotenoids [24,32], high levels of starch and fat and, therefore, high energy content (391.86 kcal/100 g) [3,5]. Studies involving the proximate composition revealed carbohydrate values ranging from 50 to 80%, represented mainly by starch [1,33]. Additionally, the fruits have high mineral content and low levels of sodium and reducing sugars [22,27,28]. Table 1 and Table 2 show recent discoveries emphasizing the carotenoids, starch, and flour from peach palm fruits. Other nutritional and functional properties, main bioactive compounds and health-promoting properties of peach palm fruit can be accessed in recent reviews [5,22,34,35,36].

In addition to direct consumption, the fruit can be used as raw material to produce many products, especially peach palm flour, oils and fermented beverages [5,47]. The peach palm in the form of flour is an alternative to avoid market saturation with fresh fruits and diversify the demand. High starch mesocarp species are the most appropriate because when starch is heated, it tends to generate technological properties of interest for certain types of products, such as bread, cakes, soups, creams, sauces and porridge [1]. The absence of gluten and starch richness makes this raw material interesting for the development of new products for patients with celiac disease [1]. Peach palm flour has been used in the production of extruded maize products and may be considered an interesting ingredient to improve the carotenoid content since the extrusion process did not affect its presence in these products [48].

### 4.2. Peach Heart

The palm heart is removed from the top of the stalk corresponding to the central part; it is soft and has a pleasant flavor and low caloric value. Another outstanding feature of palm hearts is the low activity of oxidative enzymes (peroxidase and polyphenol oxidases), reducing the risk of color change in the final product [1].

In the Brazilian market, the palm heart is sold mainly as a preserve in the form of stalks (cylinders) with a diameter between 1.5 and 4 cm. *In natura* palm heart, on the other hand, is the raw heart of the palm harvested in the field, which maintains three to four protection hems around the edible part and is 45–90 cm long. The fresh or minimally processed palm heart is the edible heart of the palm after the removal of the protection sheaths. The palm heart has a soft texture, inviting for multiple and creative gastronomic uses [49]. Table 3 shows the nutritional composition of the pupunha palm heart and juçara (*Euterpes edulis*) palm heart. Both products are rich in proteins, dietary fiber, calcium, and potassium, with low lipid content. Palm hearts are the most economically important part of the peach palm. In Brazil, the exploration of palm hearts fits as a sustainable cultivation option for small and family farming, mainly in the eastern regions of the states of Paraná, São Paulo, and Santa Catarina [8,50,51].

Twenty years ago, the Brazilian Agricultural Research Corporation (EMBRAPA)—a great state-owned company that plays a relevant role in the world’s research—implemented a sustainable peach palm production system to take advantage of areas abandoned by agriculture in the Brazilian Atlantic Forest. Today, *B. gasipaes* cultivation has a positive impact on the economic, social, and environmental aspects of several States such as Bahia, Espírito Santo, Rio de Janeiro, Paraná, and Santa Catarina. The main beneficiaries have been rural producers, who gained a source of extra income, and the palm heart agroindustry, which now has quality raw material at their disposal with a guaranteed production scale to meet market demands [52]. The incentives for the cultivation of peach palm in the South and Southeast of Brazil, with economic and environmental sustainability, reduced predatory exploitation of other species, especially of the juçara palm (*E. edulis*), native to the Atlantic Forest, which dies after the harvest of the palm. Juçara palm is a symbol of the Atlantic Forest because of its ecological, cultural, and economic value, but predatory exploitation via extraction and sale of palm hearts is seriously endangering the species [53,54]. In addition to tillering (emission of new shoots) for another 10 years, the peach palm is precocious, starting to produce palm hearts after 18 months. In overall terms, a peach palm lives, on average, twenty years, generating around 1.5 palm hearts per year. In addition, the non-oxidation of the palm heart makes it suitable for fresh sale, which makes it a source of income for small producers [8]. Currently, at least, there are also advantages for the consumer because the peach palm heart is considerably cheaper than the Juçara palm heart.

**Table 3:** Comparative nutritional composition of pupunha palm heart (*Bactris gasipaes* Kunth) and juçara palm heart (*Euterpes edulis* Martius).

Component	Pupunha Palm Heart (100 g)	Juçara Palm Heart (100 g)
Moisture	89.4%	91.4%
Energetic value	29.0 kcal = 124 kJ	23.0 kcal = 97 kJ
Carbohydrates	5.5 g	4.3 g
Proteins	2.5 g	1.8 g
Lipids	0.5 mg	0.4 g
Dietary fibers	2.6 g	3.2 g
Ashes	2.1 g	2.1 g
Calcium, Ca	32.4 mg	58.0 mg
Vitamin C	8.7 mg	2.0 mg
Phosphorus, P	55 mg	40 mg
Manganese, Mn	0.14 mg	-
Magnesium, Mg	25.5 mg	-
Iron, Fe	0.2 mg	-
Potassium, K	206.4 mg	-
Cooper, Cu	0.1 µg	-
Zinc, Zn	0.4 mg	-
Sodium, Na	562.7 mg	-

Source: TACO—Brazilian Food Composition Table [55].

## 5. Lignocellulosic Residues of Peach Palm

The production and consumption of peach palms generate a huge volume of by-products [56]. It is estimated that approximately 84% of the total weight of the palm is wasted [57]. These wastes generated in the exploration of palm hearts are environmentally and economically problematic, not to mention that they result in additional costs for the producer, who must be concerned with their proper disposal [47]. The residues generated include the peach palm fruit, which despite its biochemical importance, is not yet significantly explored [58], the stem portion (29%), the leaf sheath (17%) and the skin (37%) [48]. The use of peach palm waste fits well into the upcycling concept, which endorses the approach of recycling and reuse, thus closing the product’s life cycle [59]. The valorization of such important biomass into a spectrum of marketable products represents a promising approach for dealing with waste and, at the same time, complying with the goals of sustainable development, namely food security, environmental protection, and energy efficiency [60].

Agro-industrial waste is generally characterized by a large amount of lignocellulosic material, i.e., cellulose, hemicellulose (especially xylan) and lignin. Sources include, for example, crop residues that were primarily used for starch extraction and other food sources [61]. Forestry and pulping residues, however, are not less important in this context [62].

Cellulose and hemicellulose are macromolecules composed of sugars, whereas lignin is an aromatic polymer. Cellulose is a linear polymer composed of D-glucose subunits, which are linked by β-1,4-glycosidic bonds. The molecules of cellulose form fibrils, which are held together by hydrogen bonds and van der Waals forces. Due to these bonds, the monomers are crystalline and resistant to swelling in water and enzymatic attacks. It has been described that if water is applied at high temperature and pressure, it can break the hydrogen-bonded crystalline structure and hydrolyze the β-1,4-glycosidic bonds, resulting in glucose monomers. Hemicellulose, on the other hand, does not contain as many repeated β-1,4-glycosidic bonds and has a more random structure, resulting in a less crystalline and less resistant structure than cellulose. Hemicellulose is a polysaccharide that contains D-xylose, D-mannose, D-galactose, D-glucose, L-arabinose, 4-*O*-methyl-glucuronic acid, D-galacturonic acid and D-glucuronic acid, which are not linked solely by β-1,4-glycosidic bonds, but also by β-1,3-glycosidic bonds. Therefore, hemicellulose contains branches and is more vulnerable to hydrothermal extraction or hydrolysis than cellulose [63].

Lignin differs from cellulose and hemicellulose in that it contains aromatic rings rather than long molecular chains. Depending on the different plants and extraction processes, lignin is characterized by diverse chemical structures. In general terms, it can be considered a polyphenolic macromolecule, which consists of four monomeric units of phenylpropane, coniferyl alcohol, p-hydroxyphenyl alcohol, and synapyl alcohol. Different types of carbon-oxygen (aryl-ether) and carbon-carbon bonds are formed in different lignin subunits. As the most recalcitrant component of the lignocellulosic fibers, lignin is extremely resistant to enzymatic and chemical impacts. Lignin dissolves in alkalis and is practically insoluble in hot water, acids and a series of commonly used solvents. 

Table 4 shows the percentage of cellulose, hemicellulose and lignin from different lignocellulosic residues widely used in several technological processes. In general, the most abundant component is cellulose, followed by the hemicellulose and lignin fractions [64,65].

Lignocellulosic fibers are characterized by being abundantly available, renewable, biodegradable, and biocompatible, and they have been used in several technological applications. These properties may indicate that, with proper management, they could reduce our current society’s dependence on fossil resources. However, natural lignocellulosic materials have low solubility and processability, which limits their practical and effective use. Therefore, in general, previous treatments (pre-treatments) to remove lignin and loosen the interactions between the components of the lignocellulosic fibers are necessary to allow its subsequent use as an energy source or as a renewable resource. Physical, physicochemical, chemical, and biological pretreatments are used to make the subsequent stage of enzymatic saccharification more efficient and increase the production of fermentable sugars such as glucose.

**Figure 3:**
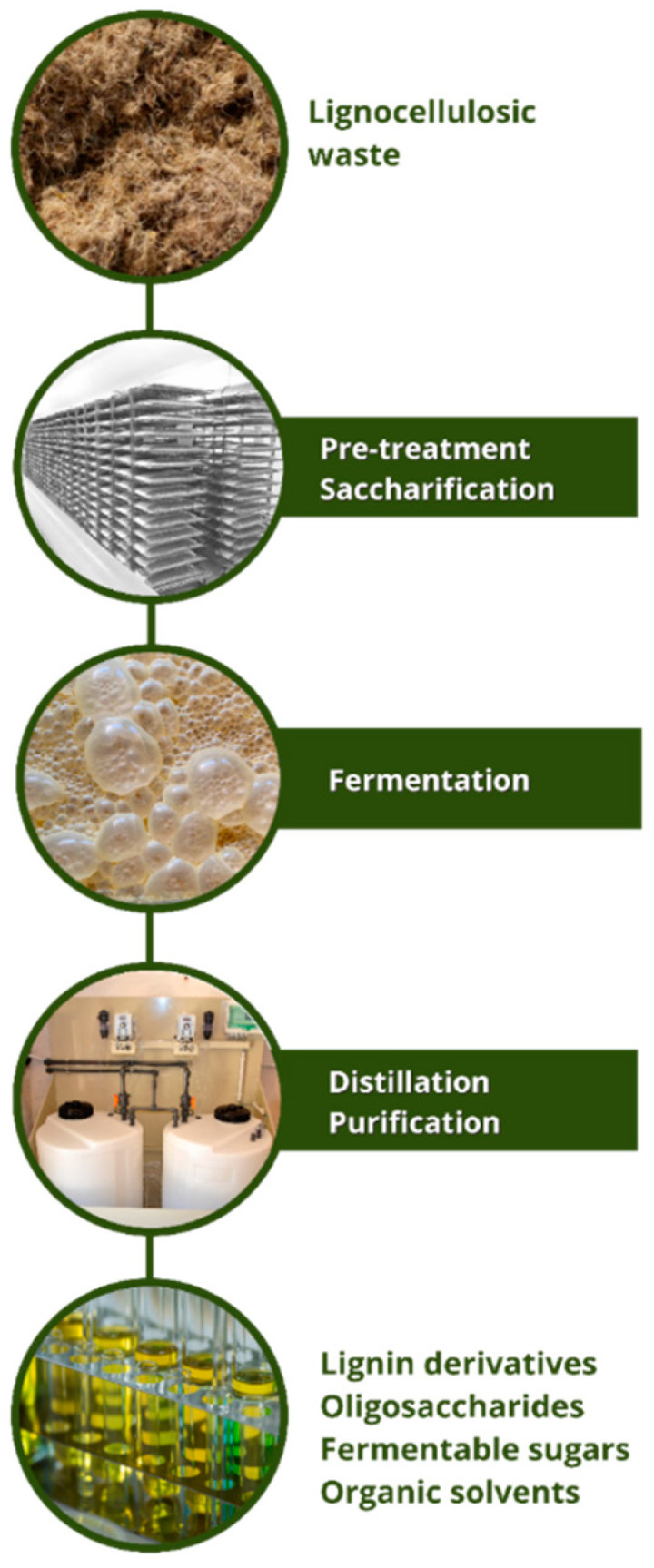
Steps involved in the bioconversion of lignocellulosic waste materials, such as those produced in peach palm exploitation, into useful high-value-added products.

In addition to pretreatment, bioconversion of lignocellulosic materials into useful high-value products requires further steps that include hydrolysis of polymers to produce easily metabolizable molecules, such as hexose or pentose sugars, and the use of these compounds to support microbial growth, either aerobically or anaerobically (fermentative processes). Finally, the products obtained must be purified by chromatographic fractionation methods or through distillation. Considering the growing demand for energy and the environmentally harmful consequences of the use of fossil fuels, lignocellulosic biomasses are the most abundant organic materials with the potential to be used in the generation of bio-based chemicals and fuels (Figure 3) [64,73,74,75].

Beyond the chemical or biological transformation of lignocellulosic residues into various products with high added value, they can also be used as substrates for the cultivation of microorganisms (bacteria and fungi). In the latter, a variety of bioactive compounds can be produced, especially hydrolytic enzymes such as cellulases and xylanases and oxidative enzymes such as laccases and peroxidases, mainly by using solid-state cultures [76,77]. Solid-state cultivations involve the cultivation of microorganisms on wet solid particles without free water surrounded by a continuous gas phase.

**Figure 4:**
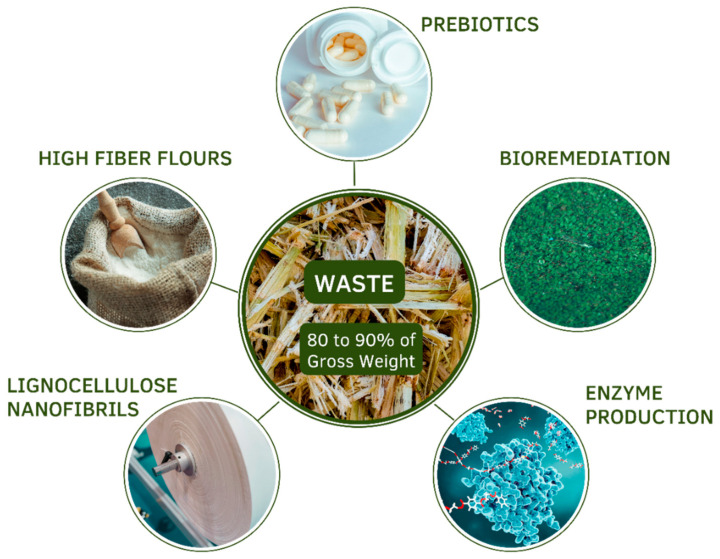
Upcycling of *B. gasipaes* lignocellulosic wastes can generate valuable biotechnological products. The median sheaths and stem parts can be processed into high fiber flours; the inner sheath and stem bark xylans are material for the obtainment of xylooligosaccharides (prebiotics); the leaf sheath can be used as a substrate for solid-state fermentation and bioremediation; the peach-palm waste can be a substrate for enzyme production, and lignocellulose nanofibrils can be extracted from the outer sheaths.

Based on the amount of waste generated with the exploration of peach palms, several studies have been carried out aiming at the use of the sheath, the stem (or bark), the fruit and the rind of the fruit. Some possible applications already carried out will be described in the following sections (Figure 4).

### 5.1. Obtaining High Fiber Flours through B. gasipaes Lignocellulosic Wastes

The interest in ingredients rich in dietary fiber has increased, and the importance of this food component has led to the development of a large market for fiber-rich products and ingredients. Dietary fibers (cellulose, hemicellulose, lignin, β-glucan, etc.) are not digested and absorbed in the human small intestine. They are threatened by complete or partial fermentation in the large intestine. Dietary fibers have beneficial physiological functions, including laxation and improving bowel health by stimulating the growth of beneficial gut micro-flora, lowering blood cholesterol and glucose levels, preventing obesity, coronary heart diseases, diabetes, blood pressure, and lowering energy intake [78]. The median sheaths and parts of the peach palm stem were evaluated for producing high-fiber flours [79,80,81]. The authors found that the flours have a high content of non-starch polysaccharides and emphasized that they are important as a source of dietary fiber for inclusion in other foods, such as breakfast cereals or bakery products. Furthermore, they observed that the flours are predominantly formed by insoluble fibers.

### 5.2. Obtaining Prebiotics (Xylooligosaccharides) B. gasipaes Lignocellulosic Wastes

Xylooligosaccharides (XOS) are composed of D-xylose units (2 to 20) joined by β(1,4) glycosidic bonds. They are mainly xylobioses, xylotrioses and xylotetraoses, which occur naturally in vegetables, fruits, bamboo shoots, milk and honey [82]. XOS can also be obtained by hydrolysis of xylans from several types of lignocellulosic biomass [83,84]. Xylans are generally branched structures that, in addition to xylose, contain arabinose (in furanose form), uronic acid (glucopyranoside form) or its 4-O-methyl derivative (2- or 3-acetyl or phenolic substituents). XOSs reach the large intestine without prior transformation in the stomach because the human body does not have the enzymes capable of hydrolyzing the β bonds. Therefore, XOSs are prebiotics that can be transformed by the colonic microbiota [25]. In addition to improving the modulation of colonic microbiota, especially bifidobacteria and lactobacilli, exerting positive effects on intestinal function, XOSs exert several beneficial effects on health, such as improved calcium absorption, protection against cardiovascular disease, decreased risk of colon cancer, reduced risk of diabetes mellitus, reduced hypercholesterolemia, immunological action, in addition to antioxidant, anti-inflammatory and anti-allergenic effects [85].

Vieira et al. [56] used alkaline extraction of xylans from the inner sheath and stem bark of peach palms. Alkaline extraction was efficient, with a yield of about 80%. Subsequently, commercial xylanase was used to obtain the XOS. The authors evaluated XOS for potential antioxidant activity compared to commercially available XOS, in addition to tentatively determining their chemical structures. The latter presented clustered units of xylose or arabinose with sodium ions adducts. In general terms, it was emphasized that the XOS from peach palm residue xylans had greater antioxidant capacity than the XOS obtained from commercial xylans. The possible exploitation of peach palm wastes to produce XOS was highlighted.

### 5.3. Obtaining Cellulose Nanofibrils from Lignocellulosic Residues of Peach Palm

Nanocellulose corresponds to the smallest part of fibers, with nanometric scales and physical properties depending on the origin of the cellulose and the production method [86]. At the nanometric level, the properties of materials are affected by the laws of atomic physics. Thus, nanocellulose has the same application potential as cellulose, but with a significantly larger specific area, with a marked improvement in its properties, especially its mechanical strength, flexibility, and thermal properties [57]. Nanocellulose has been applied in a wide range of scientific and technological areas, such as in the production of flexible films, automotive components, biomedical devices and foods, as well as in water treatment [87,88].

A combination of chemical and mechanical treatments of peach palm sheaths was used, which led to the production of cellulose nanofibrils [57]. The good dispersion stability, large aspect area and surface functional groups of these cellulose nanofibrils facilitate the interaction of the nanofibrils formed by the cellulose chains with other constituents, enhancing their potential to be used as rheological modifiers. The mechanical treatment applied by the authors improved the crystallinity (49.8–54.5%) and thermal stability of the nanocelluloses when compared to their original fibers, indicating the formation of a highly ordered cellulose structure. These characteristics, together with their high aspect ratio, indicate that they can be promising materials for reinforcement at the nanoscale.

Cellulose nanofibrils isolated from peach palm outer sheaths were recently used to improve the characteristics of cassava starch films [89]. Cellulosic material, 10 to 30 nm in diameter, was produced by mechanical defibrillation. All films were characterized by scanning electron microscopy, thermal analysis, water vapor permeability, Fourier transform infrared spectroscopy, in addition to physical characterizations (solubility, moisture, water activity, thickness, tensile strength and elongation) and optical analyses. The incorporation of cellulose nanofibrils into cassava starch films caused changes in all of the analyzed properties when compared to the control film (without cellulosic reinforcement). Physical reinforcement was the main effect observed in cassava starch films containing cellulose nanofibrils according to the analysis of mechanical strength and permeability. The spectroscopic data revealed a possible formation of crosslinking between starch and cellulose nanofibrils, which can positively influence the tensile strength of such films.

### 5.4. Application of Peach Palm Lignocellulosic Residues as Substrate for Cultivation of Microorganisms and Obtainment of Useful Molecules

Agricultural wastes or byproducts are largely used as substrates for the cultivation of microorganisms on solid-state fermentation (SSF) for the obtainment of different products. SSF is a microbial fermentation process through which selected microorganisms (bacteria, fungi and yeasts) are cultivated on a moist, solid, non-soluble organic material that acts as a support and nutrient source for the growth of the microorganisms, in the absence or near absence of free-flowing water [90]. SSF is used for the production of rich fermented animal feed, mushrooms, and important molecules such as enzymes, organic acids, antibiotics, biofuels, and secondary metabolites [91]. Wheat bran, sugar cane bagasse, rice straw and corn cob are the most used substrate for the cultivation of microorganisms. Even so, it is worth searching for new substrates, especially if they are available in large amounts, which allow the growth of microorganisms without further supplementations and facilitate the obtainment of valuable products [92,93].

A search carried out on the Web of Science found 636 scientific articles using the term sugar cane bagasse (sugar cane bagasse), 1076 scientific articles using the term corn cob (corn cob), 7024 scientific articles using the term rice husk (husk of rice) and 103 scientific articles using the term *Bactris gasipaes*, the scientific name of peach palm. Of these 103 articles, most dealt with aspects related to peach palm cultivation or the characteristics of the use of the fruit. This comparison demonstrates how, at the present time, lignocellulosic residues from peach palms are still poorly investigated in terms of their potential. Based on the studies that were described and analyzed in the preceding topics, however, the lignocellulosic residues of peach palm are likely to offer the same favorable perspectives of other lignocellulosic materials for being exploited as substrates for the cultivation of microorganisms in bioremediation processes and for obtaining high value-added products such as enzymes and prebiotics (Figure 4), as will be demonstrated by the few examples to be discussed in the next paragraphs.

The exploitation of the peel or middle sheaths of palm trees that are generated during the production of palm hearts in acid preserves for the cultivation of edible fungi has been investigated by several groups [94]. The production and commercialization of mushrooms (healthy functional foods) using peach palm residues could result in socio-environmental benefits by increasing the income of the involved individuals and by reducing environmental liability.

Lima et al. [95] studied the performance of *B. gasipaes* residues as a substrate for the growth of mycelium-based on *Lentinula edodes*. The composite formed displayed close values of other mycelium-based composites on compressive strength and elastic modulus. The authors concluded that pupunha residues are a potential alternative for mycelium-based composite. In a posterior work, the same group assessed the nutritional quality and in vivo biological activity of a *B. gasipaes* by-product food ingredient obtained via SSF by *L. edodes* [96]. Wistar rats fed with the fermented residue diet had a late and softer insulinemic peak in comparison to those of animals fed control diets (low content of protein, casein and mushroom diets). Overall, serum lipid results indicated a positive modulation of the fermented diet, which displayed good nutritional quality and the potential to positively influence glycemic and lipid profiles.

Two additional examples of research where peach palm residues were used as substrate in SSF include the cultivation of *Trichoderma stromaticum* AM7 to produce xylanases [97] and *Ganoderma lucidum* to produce ligninolytic enzymes and to decolorize an industrial textile effluent, Remazol brilliant blue dye [98].

## 6. Patents Based on *Bactris gasipaes* Products

In the past decades, a few products containing predominantly peach palm fruits and palm hearts in their formulations, or upcycled from residues of *Bactris gasipaes*, have been protected by inventors through patenting. An interesting example is plywood made out of mature trunks of peach palm, which consists of at least one plate of laths pressed horizontally by applying heat or at room temperature [99]. The inventor’s aim was to take advantage of a sub-product of the food industry (mature trunk extracted from the process of selective handling) for the production of good-quality plywood. The expectations were centered around economical availability with a high index of exploration of the raw material, dimensional stability, flexibility and mechanical resistance. Later, a patent regarding a new hydrating solid soap formulated with the active extract of *B. gasipaes*, as well as its manufacturing process, was registered [100].

More recently, a new process for the preservation of canned peach palms, which makes this food available worldwide at any time of the year, was patented [101]. Likewise, an innovative process for extracting starch from peach palm fruits based on an alkaline wet method was reported [102]. Through this process, starch granules for further applications in the field of biodegradable food and packaging can be isolated. Furthermore, methods and systems for making and using a polymer base reinforced by a powder formed from pupunha fibers were invented [103]. The resulting composite material is provided in the form of pellets for further processing.

## 7. Concluding Remarks

In Brazil, the importance of the cultivation of *B. gasipaes* (an Amazon species) to produce palm hearts has grown considerably due to its advantages in relation to other species of palms, such as precocity, rusticity and tillering. The last one is especially important, as it makes the exploitation of peach palm hearts, contrary to what happens with other palm tree species, a non-predatory practice.

The peach palm fruit is an excellent source of antioxidant bio compounds and has a superior carotenoid content when compared to the most popular tropical fruits. Its flour can be used as a functionalizing ingredient for the preparation of novel nutritious food products. However, to fully exploit the potential of *B. gasipaes* fruits towards food security, a participatory investigation is required to efficiently engage stakeholders and resolve bottlenecks regarding its industrialization and marketing stages.

Recent evidence discussed herein indicates that the *B. gasipaes* lignocellulosic wastes hold great potential for being upcycled into valuable biotechnological products. In this sense, clean technologies are protagonists of the recovery processes, ensuring the closure of the product’s life cycle in an indeed “green” way. Future research should focus on expanding and making the recovery processes economically viable, which would be of great importance for stimulating the peach palm production chain.

## Figures and Tables

**Table 1:** Recently published articles on carotenoids from peach palm fruits.

Aims and Procedures	Main Results and Implications	Reference
The ultrasound-assisted extraction (UAE) technique with various solvents [ethanol, ethyl acetate and ethanol/water (1:1, *v*/*v*)] was used to determine the best conditions for the simultaneous extraction of carotenoids and phenolic compounds from orange pulps and yellow peach palm fruits to produce extracts with high bioactive compound contents.	Regardless of the solvent, the UAE proved to be an efficient technique to carry out simultaneous extraction of high contents of carotenoids and phenolic compounds from pulps of peach palm fruits.	[37]
A new form of extraction mediated by ionic liquids (ILs) was used for the extraction of carotenoids of *B. gasipaes*. Four ILs were examined, as well as the solid-liquid ratio *R*_(S/L)_, the number of extractions, the time of extraction, the co-solvent-ratio *R*_(IL/E)_ and the homogenization method employed.	After selecting the best solvent ([C_4_mim][BF_4_]) and process conditions (extraction yield of 172 ± 18 μg carotenoids g dried biomass^−1^), the IL-ethanolic solution recyclability was tested by freezing/precipitating the IL (maximum of 94% of IL recovered), proving its success for at least 10 cycles while decreasing the process carbon footprint by 50% compared with the conventional method using acetone.	[38]
The extracts of *B. gasipaes* rich in pro-vitamin A carotenoids were emulsified and subjected to an in vitro digestion model followed by the Caco-2 cell absorption assay.	The cellular uptake of the carotenoids extracted with ionic liquid was 1.4-fold higher than that of those ones extracted using conventional organic solvents.	[39]
Carotenoids from *B. gasipaes* waste obtained by an ionic liquid (IL)-based process were investigated in terms of safety, anti-inflammatory and antioxidant activity on the kidney of high-fat-diet (HFD) animals. Wistar rats were supplemented or not by carotenoids extracted with IL or deep eutectic solvents (VOS).	The animals supplemented with carotenoids had lower weights than the controls and the high-fat diet group. In the animals supplemented with carotenoids, the IL group had improved anti-inflammatory and antioxidant activities compared with the group supplemented with carotenoids obtained by VOS. Also, the HFD-VOS group showed moderate-severe injuries in the kidney. ILs could represent a novel tool for natural pigments safely applied to the food industry.	[40]
Response surface methodology was used to investigate the effect of process variables on the ultrasound-assisted extraction (UAE) of total carotenoids from peach palm fruit with sunflower oil.	The optimal UAE condition was obtained with an ultrasonic intensity of 1528 W/m^2^, extraction temperature of 35 °C and extraction time of 30 min. Under these conditions, the maximum extraction of total carotenoids was 163.47 mg/100 g dried peel.	[41]
This work proposed to investigate the potential of carotenoids extracted from *Bactris gasipaes* feedstocks, mediated by an ethanolic solution of an imidazolium-based ionic liquid (IL). Male Wistar rats were randomized into six different groups, supplemented or not by carotenoids extracted by IL or deep eutectic solvents (VOS) and fed by control- and/or high-fat diets (HFD). The adipose tissue-liver axis was studied as a model to investigate the influence of carotenoids on the levels of inflammation and oxidative stress markers.	The main results showed that animals supplemented with carotenoids extracted with IL displayed improvements in serum parameters, besides lower metabolic efficiency, and improved antioxidant response in the liver, even when fed with HFD. However, animals supplemented with carotenoids extracted by VOS showed higher levels of pro-inflammatory markers and pronounced oxidative stress in the liver.	[42]
Extracts obtained from peach palm fruit using supercritical carbon dioxide were compared in terms of yield, total phenolic content, total flavonoids, total carotenoids, and antioxidant activity by means of the β-carotene bleaching method.	The recommended operating condition for supercritical extraction was 300 bar–40 °C because this allows for obtaining the highest carotenoid concentration.	[43]

**Table 2:** Recently published articles on starch and flour from peach palm fruits.

Aims and Main Results	Implications	Reference
Water-extracted starch from the mesocarp of peach palm fruit presented small granules with a smooth surface and oval or conical shapes and larger spherical granules with holes and cracks on the surface. The amylose content was less than 20%, and the amylopectin revealed a crystalline structure with a high proportion of medium chains (13–24 residues). X-ray diffraction suggested a low digestible starch. The crystals were apparently homogeneous, and a weak gel was formed after 24 h of storage.	The peach palm fruit starch can be used in products where slow and smooth retrogradation is desired, such as in bread, soups, chowder, and porridges, without the use of emulsifiers or fat.	[3]
Techno-functional properties of the flours from two ecotypes of peach palm fruit peels were evaluated. Temperature and particle size had a pronounced influence on most of these properties except the swelling capacity. The flour from the red ecotype revealed superior nutritional properties in terms of total dietary fiber and protein contents and was also superior in terms of its water retention capacity, oil retention capacity, emulsifier activity and emulsifier stability.	These results suggest that by considering its protein contents and the fact that it can be considered as a source of dietary fibers or emulsifiers, the flour from the peach palm fruit peels could be used as a promising natural additive by the food industry.	[44]
The possibility of producing cookies using flour from peach palm fruits was investigated. Analysis ranged from composition, physicochemical properties and hygroscopic behavior. Cookies produced with two types of peach palm flour, whole fruit (pulp + peel) and solely pulp, presented good sensory acceptance (>70%), but the purchase intention favored the cookies prepared with whole fruit flour (85%). Both types of cookies (whole fruit-pulp) presented low moisture (4.9–6.2%), high lipid content (25.56–26.37%) and total carbohydrates (59.10–61.84%), resulting in products with high total energetic value (501.8–502.8 kcal/100 g).	The authors concluded that peach palm peels represent an excellent alternative for the use of by-products in the development of new food products	[45]
Pupunha flours (PF) from fruits harvested at different locations were characterized concerning their phenolic contents, cytotoxic effects, and inhibition of protein digestion in vitro. PF has high contents of phenolic compounds and antioxidant potential.	Potential negative effects such as cytotoxicity cells and inhibition of protein digestion in vitro were described for the first time and related to the high contents of phenolic compounds in the flours.	[46]

**Table 4:** Cellulose, hemicellulose, and lignin contents of lignocellulosic residues used in the generation of high-value-added products.

Lignocellulosic Biomass	Dry Weight (%)			Reference
	Cellulose	Hemicellulose	Lignin	
Bamboo	45.8	26.6	23.4	[66]
Corn stalk	27.8	10.1	34.1	[67]
Corn husk	48.6	16.1	6.5	[68]
Oat hull	38.7	35.3	10.1	[68]
Wheat straw	35.5	25.2	25.1	[69]
Sugar cane bagasse	58.8	17.7	12.7	[70]
Eucalyptus sawdust	42.0	19.0	23.0	[71]
Rice husk	49.6	10.4	21.8	[70]
Rice hull	35.1	26.3	20.0	[72]
Peach palm	34.2	21.3	19.5	[57]

## Data Availability

Not applicable.
